# Metagenomic Studies in Inflammatory Skin Diseases

**DOI:** 10.1007/s00284-020-02163-4

**Published:** 2020-08-19

**Authors:** Urszula Godlewska, Piotr Brzoza, Kamila Kwiecień, Mateusz Kwitniewski, Joanna Cichy

**Affiliations:** Department of Immunology, Faculty of Biochemistry, Biophysics and Biotechnology, Jagiellonian University, Kraków, Poland

## Abstract

Next-generation sequencing (NGS) technologies together with an improved access to compute performance led to a cost-effective genome sequencing over the past several years. This allowed researchers to fully unleash the potential of genomic and metagenomic analyses to better elucidate two-way interactions between host cells and microbiome, both in steady-state and in pathological conditions. Experimental research involving metagenomics shows that skin resident microbes can influence the cutaneous pathophysiology. Here, we review metagenome approaches to study microbiota at this barrier site. We also describe the consequences of changes in the skin microbiota burden and composition, mostly revealed by these technologies, in the development of common inflammatory skin diseases.

## Introduction

**Fig. 1:**
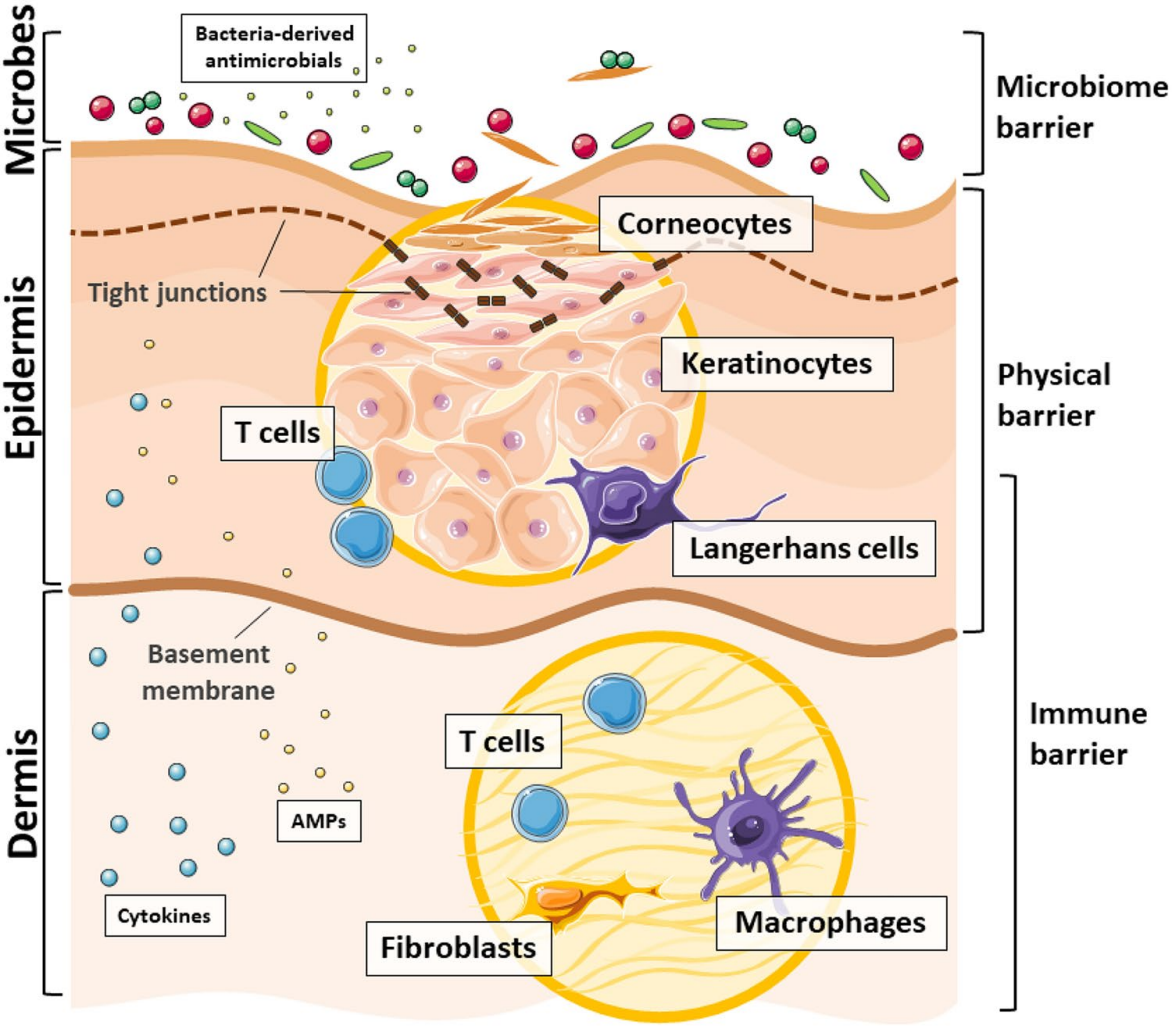
Skin is the first line of defense against various microorganisms. In order to achieve its primary function, skin consists of different layers, starting with microbes on the skin surface. These various bacteria create microbiome barrier, which secrete antimicrobial factors and contribute to the skin defense mechanisms. Keratinocytes in the epidermis, which constantly divide and differentiate into corneocytes, are a vital part of the skin physical barrier. These cells create a solid layer, interconnected by tight junctions, that faces the environment and holds back microorganisms. Shedding of the dead skin cells also helps to limit the number of microbes on the skin. Finally, the immune barrier in the dermis consists of different immune cells, which altogether with keratinocytes and fibroblasts sense the danger signals and produce various cytokines and antimicrobial peptides

Skin is the largest human organ with a primary role in isolating body from the external environment (Fig. 1). The cutaneous barrier is a physical obstacle made up of dead, superficial keratinocytes (called corneocytes), and tight junctions that confer protection against environmental factors. Barrier integrity is also supported by immune cells, a plethora of antimicrobial peptides (AMPs), various soluble mediators, or low pH. In response to microbial stimuli, almost each cell type in the skin can participate in immune defense. Keratinocytes, fibroblasts, and sebocytes can secrete inflammatory mediators that support activation and migration of immune cells. That includes cytokines, chemokines, growth factors, proteases, and other agents modulating both innate and adaptive immune responses [1]. Acidic environment is necessary for the maintenance of antimicrobial protection of stratum corneum, the most superficial layer of the epidermis. Acidic pH creates suitable conditions for limiting growth of certain microorganisms, as well as provides appropriate environment to the synthesis and processing of epidermal lipids catalyzed by pH-dependent enzymes [2]. Lipids synthesized in epidermis and sebaceous glands, together with various skin-derived AMPs, protect skin from a broad range of microorganisms. These elements constitute a highly effective line of defense against pathogens [1] (Fig. 1).

The cutaneous microbiome forms one of the largest microbial ecosystems in the human body and is recognized as an essential contributor to the skin barrier function by regulation of microbial composition of the skin and function of immune system [1]. Skin residents shape microbial communities through various strategies. These consist of competition for nutrients and space [3], production of antimicrobial factors [4, 5] and stimulation of secretion of host-derived AMPs [1, 6]. All that restricts growth of more invasive species. Moreover, microorganisms can amplify immune responses via activation of toll-like receptors (TLRs) [7] that lead to secretion of immune mediators (like cytokines, AMPs) [1, 8]. In addition, they are also involved in establishment of immune tolerance [9]. Given growing evidence that the skin microbiome is an integral component of healthy skin environment, any modifications in the composition of the microbial communities may contribute to a loss of a gentle balance in the skin and subsequently promote the development of skin diseases [10, 11].

In the past, all studies conducted to characterize microbial content of skin tissue had to rely on traditional culture-dependent techniques. However, such an approach overlooks a high number of unculturable microbes. These can be readily detected nowadays using a metagenomic approach. This broad term refers to using modern genomic techniques for sequencing a large set of genes within a sample without a need for isolation of individual species [12]. Metagenomics enables deeper exploration of microbiome diversity and characterization of “microbial dark matter”—a collection of unknown species that cannot be cultured [13]. These approaches can lead not only to more comprehensive identification of bacterial species residing on skin but also reveal microbial response to specific stimuli at transcriptome level [14]. A number of conducted metagenomic studies have risen rapidly over the past 5 years. However, there are several drawbacks associated with this technology. Researchers are confronted with a robust array of computer tools for analyzing their data. Lack of clear guidelines limits conducting high-throughput experiments as both environmental factors, sample processing and analysis methods can influence results. Therefore, findings stemming from metagenomic studies should be read with caution, especially if they stand in opposition to other results.

In this review, we briefly summarize the current NGS methods employed in metagenome studies as well as discuss recent findings on implications of changes in cutaneous microbiota on skin diseases. Here, we focused on three inflammatory disorders: acne vulgaris, atopic dermatitis, and psoriasis, in which specific microbial disturbances play important role in pathogenesis. Understanding how various skin disorders can be linked to the composition of cutaneous microbiome can help to modify current therapeutic strategies by implementation of probiotics, novel antimicrobials, or even microbiota transplants [15, 16].

## Studying the Metagenome—Techniques and Approaches

Before emergence of NGS methods, the primary technique to study interactions between microbial species was based on culturing a limited set of microbial taxa in vitro [17, 18]. Such an approach, however, faced many issues. A vast portion of microbes are unculturable, with others growing at rates different than those in situ*.* Therefore, these studies were both difficult to conduct and prone to errors [19].

Most recently, thanks to the advent of high-throughput sequencing techniques, usage of bacteria culturing in metagenomic studies is no longer necessary. The experimental weight has moved from culture method choices to proper sample collection, most adequate technique choice as well as unflawed raw data processing and results visualization. Several different strategies can be employed in metagenomic studies, each with a different set of advantages and disadvantages.

**Fig. 2:**
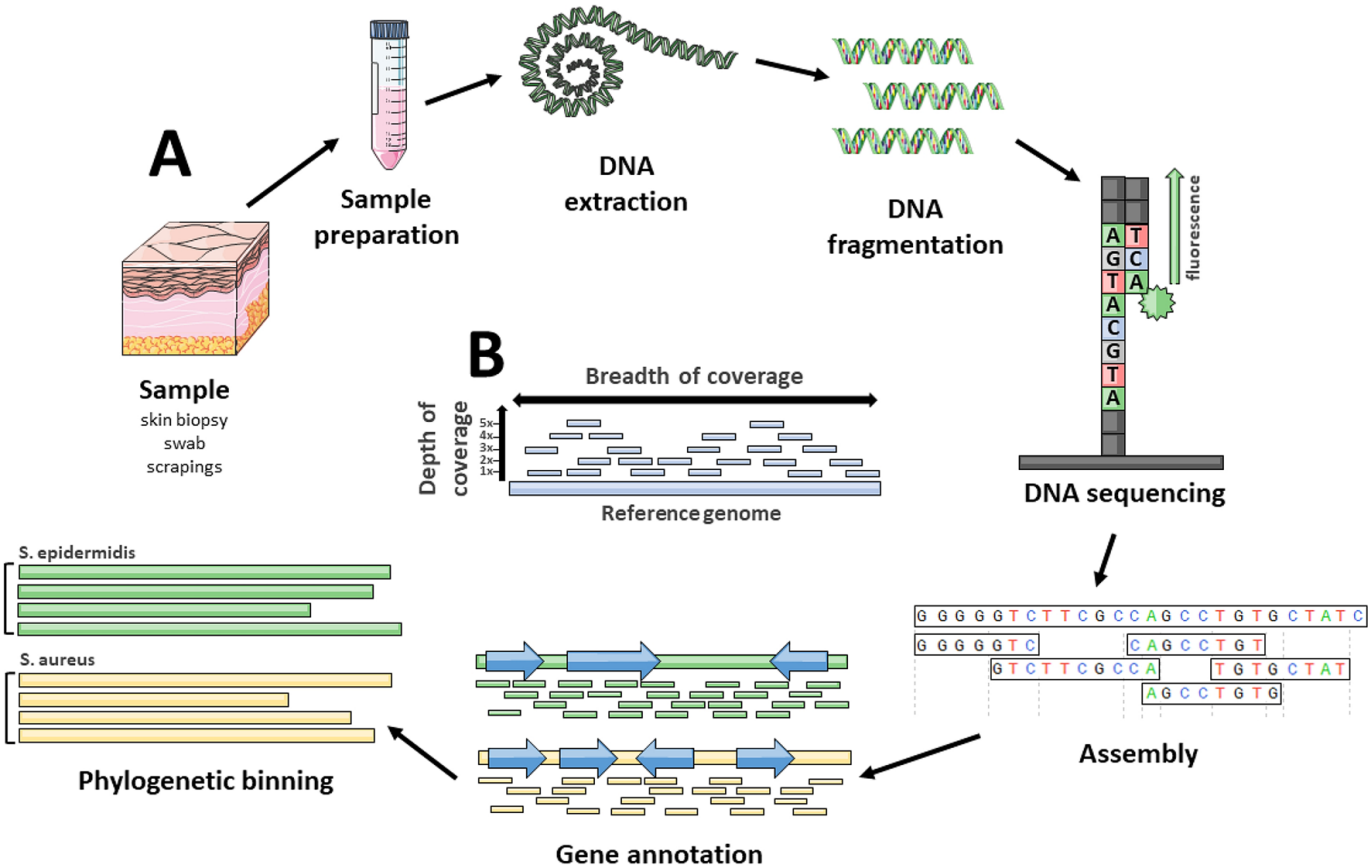
Overview of methods used in a typical whole shotgun (meta)genomic sequencing study. **a** After sample collection and DNA extraction, genetic material needs to be fragmented. Generated short DNA fragments are all sequenced in parallel in the following step (sequencing by synthesis depicted, characteristic, e.g., for Illumina method). Resulting short reads need to be assembled in silico to create sufficiently long segments for gene annotation and/or whole-genome assembly. For this, either database reference genome is used, or de novo assembly is conducted. In the next step, a variety of procedures can be undertaken, including phylogenetic binning of resulting sequences. For the assembly to succeed, sufficient representation of genomes (depth of coverage) must be achieved within the short fragment (**b**). Given high sample complexity (breadth of coverage), this aspect is especially important for metagenomic studies

Currently most widely used sequencing methods employ DNA amplification and library preparation of short (usually no longer than 300 bp) amplified segments [20] (Fig. 2). Those are then sequenced either by synthesis (SBS) or ligation [21]. In case of shotgun sequencing, sample DNA is first fragmented to achieve desired length. A sufficient representation of amplified fragments (depth of coverage) must be achieved in order for computer algorithms to successfully reassemble the sequences of interest [22]. Then, the sequenced data need to be further assigned into bins (binning) corresponding to their taxon ID in order to get the taxonomic diversity profile of a sample. Consequently, very similar sequences can be difficult to distinguish, mis-binned, or can be misidentified as sequencing errors. Notably, in contrast to gut microbiota, studies of cutaneous microorganisms are hindered by considerable contamination with host material. Given a relative low abundance of microbial material on the skin, up to 90% of reads can originate from human cells.[23]. To combat this obstacle, enrichment techniques can be utilized during amplification or library creation [24, 25].

## NGS Methods for Metagenomics Studies

### 16S rRNA Sequencing

Specific targeting of variable regions of 16S rRNA gene has long been a useful method in identifying bacterial species. Similar approach can be conveyed using NGS techniques. While parts of 16S rRNA gene are conserved, others, named hypervariable regions (denoted V1-V9), can act as phylogeny markers. 16S rRNA sequencing is one of the least expensive metagenomic approaches. In this method, primers targeting conserved sequences next to variable regions are used to amplify the variable parts. Amplicons are then sequenced, and the resulting reads are aligned to reference taxonomy database such as NCBI Taxonomy Database [26], SILVA [27], or Ribosomal Database Project (RDP) [28]. Notably, downstream results can depend on the choice of reference database [29]. Fungal composition can be studied in a similar way, with the Internal Transcribed Spacer 1 (ITS1) region as a target [30]. 16S rRNA sequencing uses a specific set of primers which minimizes the problem of a sample contamination. This method, however, can be prone to bias on multiple levels. First, more abundant species tend to be overrepresented, to a point where rare ones are hard to detect [31]. Moreover, depending on the choice of primers and sequenced regions, the results can be further skewed [32]. Additionally, 16S rRNA sequencing requires prior knowledge of the microbial community (or, more precisely, raw result must match a sequence in a database for species identification). Lastly, the resolution is usually restricted to genus level and the amount of functional information provided by the analysis is limited [33]. All that makes targeted sequencing a first-choice method, especially for studies involving screening of microbiota composition.

### Whole-Genome Shotgun Metagenomics (WGS)

Unlike the previous approach, shotgun metagenomics does not target any specific sequences (hence “shotgun”) and aims to sequence all genomic material within the sample. It enables simultaneous study of the whole microbiome, including myco- and viro- biome. Amount of information can be sufficient for a strain-level resolution. Also, given an adequate depth of analysis, extracting functional information is also achievable [34]. These vary depending on the aim of the study e.g.: from relative abundance of specific microbial species, search for novel genes in specific environments [35], to studying emergence of antibiotic resistance [36]. While WGS offers a greater insight into the microbiome, there are multiple challenges that need to be overcome. For instance, samples with low microbial content – such as skin swabs – often require enrichment methods to be employed as the vast majority of genetic material in such samples comes from the host, not the microbial cells. Without enrichment, metagenomic data analysis is much more computationally difficult and costly, if possible at all. These techniques are often based on selective lysis of eukaryotic cells during DNA isolation, or more sophisticated sorting of isolated DNA based on its specific traits—such as methylation pattern [37]. Such enrichment, however, can on its own introduce bias into sequencing data [38]. In contrast, gut microbiome samples usually need no enrichment as the bacterial content is high enough. Shotgun sequencing is much more compute- and resource- intensive than a targeted approach. For data analysis various pipelines can be applied, usually using a mixture of de novo (prioritizing whole-genome assembly from sequenced data) or read-based profiling (where reads are compared to database sequences) [39]. Given data complexity, analysis tools are rapidly evolving, with novel approaches such as machine learning being employed [40]. Vast amount of information generated and superior resolution make this the preferred method for elucidating high-complexity microbial communities or for comparative studies. Some of the WGS problems might be resolved as the third generation of sequencing methods become more prevalent. For example, Pacific Biosciences’ or Oxford Nanopore Sequencing do not rely on amplification methods and instead offer single molecule sequencing. This in turn overcomes the short read limit and problems emerging from repeated sequence elements [41]. Consequently, the genome assembly is less compute-intensive given it is easier to create a continuous genome assembly (contig) from longer fragments. These methods, however, are still characterized by a higher error read rate (a chance that a single nucleotide was misidentified) than 2nd generation methods [42], requiring use of specific data processing that account for that fact while potentially making them unviable for certain applications such as clinical diagnostics [43].

### Transcriptomics

Both targeted sequencing and metagenomic shotgun approach focus solely on the DNA content of the studied sample. While this enables a detailed study of microbial phylogeny or most prevalent gene families, it carries little information about functional aspects. Metatranscriptomic approach instead focuses on sequencing the gene transcripts. On a most basic level, it provides insights into the sets of genes that are undergoing expression. Similar to a classical RT-qPCR approach, relative expression or changes in expression can also be studied. Like all other NGS methods, this approach has its shortcomings. Prokaryotic mRNAs are notoriously short-lived, making difficult to “freeze” an actual expression profile [44]. Furthermore, lack of a specific sequence (like a poly-A tail) complicates amplifying all transcripts without any bias [45]. Contamination with host material can also pose a problem. Most of all, assigning specific transcripts to specific bacterial species may prove impossible as appropriate databases are limited. Despite such challenging characteristics, metatranscriptomic approach is becoming more widely used [46, 47], even in the context of skin microbiome [14]. This novel approach allows for an in-depth host-microbiome interactions, specially in pathological conditions. On the other hand, sequencing of host mRNAs is already relatively easy and has been successfully used in a myriad of studies, for example, focused on host response to microbial colonization [48].

### Bioinformatic Approaches to Data Analysis

Another challenge of NGS studies is data analysis. Sole number of sequencing reads does not allow for manual inquiry, most notably a quality control. A series of operations is performed in sequence on the input data that together form what is referred to as a pipeline. A well-constructed pipeline is a foundation of a successful NGS experiment. The general steps involve: trimming and excluding low-quality reads, assembly of short reads into overlapping, consensus sequences (contigs), alignment to database sequences, phylogeny and/or functional identification, statistical analysis, and visualization [49]. From a practical standpoint, the implementation of pipeline design (how a researcher selects each step in data manipulation to achieve his/her needs) ensures a successful data analysis. Several methods to pipeline design have arisen. Each approach offers its pros and cons.

The most skill-intensive method involves custom pipeline assembly in languages like R or Python (and Linux environment) [50]. This method offers the greatest level of customization, including custom-made scripts, potentially offering the most precise answers to research hypothesis. However, there are also a number of shortcomings associated with this approach. Pipeline handling, customization, and maintenance—require skilled bioinformaticians to handle updating. Furthermore, as team members rotate, it may become increasingly hard for new team members to maintain the custom solution, potentially leading to a need of a thorough rewrite. While the software used in this approach is, for the most part, free from any license fees, the hardware (a workstation-class computer or an access to a computational shard) may constitute a major expense. Finally, custom approach is the most prone to errors in a standardization of data analysis, making it hard to reproduce results of other groups [51].

Another approach involves non-commercial packages like Quimme2 [52] or Galaxy [53]. These packages allow for a more streamlined data manipulation. They often offer graphical user interface or ability to work from a web-browser level. Furthermore, most use readily available servers for data processing, making those tools virtually free to use. Notable downsides involve less room for customization, often limited pipeline selection, and limited space for data storage. Moreover, data processing may take a long time depending on existing server load time. Lastly, attractive data visualization often requires additional software tools to be employed, usually requiring at least basic knowledge of programming languages like R or Python.

As a response to the NGS boom, several commercial analysis platforms have emerged, with examples like Explify® [54] or Qiagen CLC Genomic Workbench [55]. These offer the most streamlined experience for a non-informatician, with relatively easy to setup pipelines, graphical user interface, data visualization suite and potentially professional support. While the advantages are clear, the downside is a relatively high cost, often calculated on a per-analysis basis.

Given a tremendous amount of information generated in each NGS experiment, several notions of data storage and reusability have arisen. Several repositories are available like the NCBI Sequence Read Archive [56] or the European Bioinformatics Institute MGnify [57]. Reusing such datasets for further studies has been a major goal of novel scientific guidelines e.g.: the European Commission FAIR ecosystem [58].

### NGS in Skin Microbiome Research

Over the past decade, largely as a result of metagenomic revolution, an important role of the microbiomes on regulation of pathophysiological processes at barrier sites has emerged. The human microbiome research is mostly focused on the microorganisms colonizing the digestive system, where bacterial composition has been linked to multiple pathologies, such as inflammatory diseases, allergies, diabetes, obesity, cancers and depression [59]. Nevertheless, understanding the role of the cutaneous microbiota can shed a new light on variety of inflammatory skin diseases. Deciphering the composition of healthy cutaneous microbiome serves as a first step to explore mutual interactions between microbes and the host. Skin microbiota consists primarily of bacterial, fungal and viral communities, with bacteria being the best characterized component of the human microbiome [60]. Healthy skin is predominantly colonized by bacteria from four phyla; *Actinobacteria, Proteobacteria, Firmicutes* and *Bacteriodetes* [3, 23, 61]. Bacteria mainly reside on the skin surface or within skin appendages [62, 63]. Because the distribution of appendageal structures differs across skin regions, so does the abundance of cutaneous bacteria. Moreover, chemical characteristics of these sites, such as an oily microenvironment of sebaceous glands, or a moist niche of sweat glands, favors growth of certain microorganisms over others, influencing taxonomic composition of bacterial communities across skin regions. For example, sebaceous sites are preferentially inhabited by *Cutibacterium* (formerly *Propionibacterium*) species, whereas *Staphylococcus* and *Corynebacterium* populate moist areas of the skin [61, 64, 65]. Common dermatological disorders preferentially appear in specific skin sites, depending on chemical landscape of skin. For instance, atopic dermatitis tends to manifest on the inner bend of the elbow (moist region), whereas psoriasis on the outer part (dry region). For that reason, characterizing the composition of microbial communities in specific skin areas should provide better understanding of mechanisms that drive dermatological disorders [8, 61].

### Alterations of Microbiota in Skin Diseases

Skin is a heterogeneous ecosystem, where uneven distribution of glands and hair follicles creates, physiologically and topographically distinct, complex niches that are home to a wide range of microbes, preferring a variety of living conditions. In healthy skin, microbiota harmoniously co-exists with the host. Under homeostatic conditions, well-balanced microbial communities support tissue health, while breakdown of this “peaceful” composition of microorganisms disrupts homeostasis, including immune regulation. As a result of alterations in microbiota burden and composition (referred to as a dysbiosis), altered immune responses might contribute to pathogenesis of skin diseases like psoriasis or atopic dermatitis [8, 66, 67]. For example, well-known pathogens, such as *S. aureus* inhabit the healthy skin often without much harm. Nonetheless when the epidermal barrier and/or well-balanced microbial composition is disrupted, the bacteria become more harmful [8]. The analysis of the most common cutaneous species between individuals showed strain-level variation [3, 65]. Phenotypic variability of the most abundant species is known to change in compromised skin [3, 60, 65, 68]. In such conditions typical skin commensals like *Cutibacterium acnes* or *Staphylococcus epidermidis*, may show their contextual pathogenicity. For instance, *S. epidermidis* is usually associated with positive outcomes for the host health through interactions with keratinocytes, immune cells and skin microbiota. This interplay promotes skin health by reducing inflammatory response, limiting a bacterial infection and stimulation of wound repair [69]. However, *S. epidermidis* represents common source of nosocomial sepsis, as a result of contamination of medical devices. Most infections are caused by methicillin-resistant *S. epidermidis*, encoding peptide toxin PSM-mec that is equipped with sepsis-associated proinflammatory and cytolytic properties [70]. Likewise, C. *acnes* exhibits two-faced behavior, either protecting against host pathogens or leading to pathogenesis of acne vulgaris [71]. While it still remains obscure why and how benign bacteria can switch to aggressive behavior, this behavior may be attributed to the horizontal gene transfer (HGT). Through distinct mechanisms, including conjugation, transduction or transformation, HGT is a source of genetic diversity for bacterial communities. For example, typical genes of staphylococcal virulence *ssaA* and *esxA* as well as markers of HGT (transposases, integrases, and other phage-related genes) were found in psoriatic lesions. This phenomenon may help to explain mechanisms of transition in contextual pathogen behavior in the compromised skin [11].

On the other hand, whether imbalance in microbial composition is the cause or the effect of a disease is not well understood. Although still hypothetical, host factors, including AMPs, might be a potential missing link that can facilitate dysbiosis-dependent skin illnesses. AMPs serve as the first line of immune defense, sharing both antimicrobial and immunomodulatory properties. In skin, some of AMPs such as human β defensin 1 (hBD1) and RNase7 are constitutively expressed to create antimicrobial barrier, while others are only induced in certain conditions, such as inflammation, infection, or injury. Specific upregulation of host AMPs in response to skin-associated bacteria seems to be beneficial for maintaining skin homeostasis via a direct antimicrobial action or a stimulation of immune response [72, 73]. However, overexpression of certain AMPs with immunomodulatory properties can trigger excessive inflammatory response. For example, peptide LL37, one of the best characterized member of the cathelicidin family, can be responsible for breaking tolerance to autoantigens through its ability to sense nucleic acids [1, 74]. Antimicrobial agents derived from psoriatic scales that include LL37, hBD2, and hBD3 are able to induce nucleid acid-dependent activation of plasmacytoid dendritic cells (pDC) and therefore contribute to the pathogenesis of psoriasis [75]. In contrast to psoriatic skin, the content of specific AMPs, such as cathelicidins, hBD2, and hBD3, are reduced in atopic dermatitis. Innate antimicrobial barrier is postulated to be impaired by the Th2-derived cytokines that suppress the production of AMPs. Consequently, it facilitates extensive *S. aureus* colonization of atopic skin [76, 77]. In acne, the upregulation of various AMPs promotes an excessive immune response that worsens skin condition [78].

### Acne Vulgaris

Acne vulgaris (commonly called acne) is highly prevalent inflammatory disease of pilosebaceous unit (hair follicles and their accompanying sebaceous gland), characterized by comedones and pimples. Multiple factors are involved in pathogenesis of acne, involving abnormal sebum production, hyperkeratinization, and proinflammatory response to *C. acnes* [78]. *C. acnes* is one of the most abundant lipophilic species in sebaceous sites of the skin, capable of mediating skin protection against harmful microbes. For example, the free fatty acids generated by *Cutibacterium* spp. modify the pH and act directly as antimicrobial factors. *C. acnes* is thus regarded more likely as a beneficial cutaneous species that primarily promotes skin health [71, 79]. Nevertheless, it has been considered to be a pathogenic factor in acne development for more than 100 years [80]. Recent studies have facilitated our understanding of the role of *C. acnes* in the pathogenesis of acne. Comparison of 16 rRNA collected from hair follicles of acne patients and healthy individuals demonstrated that relative abundance of *C. acnes* was similar in both groups of donors [81]. Another analysis of skin microbiome based on ultra-deep metagenomic shotgun sequencing identified more *C. acnes* in the follicular microbiome of healthy individuals. In the same study acne patients were shown to have a slightly lower relative abundance of *C. acnes* and related *C. granulosum*, whereas minor bacterial taxa were more prevalent [68]. Many conditions, including hypoxia or altered lipid metabolism, might influence host-microbiome interplay within the follicular microenvironment that subsequently drive the disease progression [78]. Based on these, it was proposed that both, inflammation and reduction of *Cutibacterium* abundance can modify lesional skin in a way that favors colonization by non-commensal species [68].

As was referred previously, diversity at strain level might be associated with more harmful phenotype of species normally considered as benign. Strain-specific sequence differences were apparent when comparing *C. acnes* genome from affected and healthy individual samples [81]. Genome analysis of *C. acnes* showed that isolates from acne-suffering patients contain plasmid and chromosomal regions encoding a number of genes involved in virulence [68, 81]. At the same time genomes derived from normal skin samples were instead enriched in unique genetic elements encoding Clustered Regularly Interspaced Short Palindromic Repeats (CRISPR) and Cas proteins [81]. The CRISPR/Cas mechanism is well known to guard bacteria against invasion by other foreign DNA, including phages and plasmids [82]. Together, those findings suggest that specific genetic elements determine passive and aggressive behaviors of commensal-like *C. acnes* [81]. In summary, acne vulgaris is the flagship example of a disease, where commensal bacteria can become pathogenic. Although the causes of pathogenic behavior of *C. acnes* are still largely unknown, recent findings uncovering the previously uncharacterized host–microbe interactions and strain-specific virulence determinants can help in better understanding of the role of *Cutibacterium* in acne.

### Atopic Dermatitis

Atopic dermatitis (AD, atopic eczema) is a chronic inflammatory skin condition in which the epidermal barrier is impaired. AD pathogenesis has been attributed to complex interactions among genetic, environmental, immunological, and microbial factors that trigger Th2-mediated skin inflammation [83]. The decreased diversity of microbial community on the skin surface along with overrepresentation of *S. aureus* are considered as key determinants of AD [83–85]. Depending on the disease state, specific bacterial shifts were found in both non-inflamed and inflamed AD skin. Examination of non-inflamed skin samples from AD-susceptible individuals showed elevated representation of *Streptococcus* (including α-haemolytic species)*, Gemella, Veillonella* and *Haemophilus*. The majority of these species are known as components of commensal oral microbiota, but under certain conditions can act as opportunistic pathogens. The impact of these species on AD pathology remains unknown; nonetheless, they could play a role in sensitizing the immune system in the skin [86]. AD severity was associated with predominance of *Staphylococcus* species, particularly S. *aureus* and S*. epidermidis* [60, 66]. *S. aureus* was the abundant species in severe AD, while *S. epidermidis* was found in patients with mild to moderate AD. Inflamed AD skin was colonized with a single clade of *S. aureus*, in contrast to heterogeneous communities of *S. epidermidis* in individuals with a less severe AD [60]. Dominance of *S. aureus* in AD subjects was correlated with decreasing abundance of coagulase-negative staphylococci that are capable of producing antimicrobials against *S. aureus* [5]*.* Analysis of microbiota collected from infants showed that early skin colonization by commensal staphylococci might protect from development of AD [87]. *S. aureus* harbors numerous components that enhance adhesion to skin surface or damage the epidermal barrier. This binding is mediated by the bacterial adhesins that interact with host fibronectin and fibrinogen [83, 84]. Overgrowth of *S. aureus*, as well as its toxins could induce immune response through T cells, macrophages, and mast cells' activation [84]. Of note, enhanced *S. aureus* skin colonization is correlated with upregulation of Th2 cytokines -a characteristic feature of atopic inflammation. Analysis of cytokine profile shows that clinical isolate of *S. aureus* from AD skin shifts the Th1/Th2 balance toward Th2 response, whereas laboratory strain of *S. aureus* or *S. epidermidis* are more likely to stimulate Th1 response [88]. Taken together, these datasets suggest that in atopic skin, *Staphylococcus* spp. play opposite roles—exacerbating disease symptoms or inhibiting AD pathogenesis. Collectively, these studies confirm the overrepresentation of *S. aureus* as a key bacterial microbiota component involved in the pathogenesis of atopic dermatitis.

### Psoriasis

Psoriasis (PS) is a chronic inflammatory skin disease characterized by keratinocyte hyperplasia, altered keratinocyte differentiation, as well as a dense skin leukocyte infiltrate. In contrast to AD, where Th1/Th2 balance is shifted toward Th2-type immune response, the development of psoriasis is mediated by aberrant activation mostly of Th17 cells and also Th1 cells [89, 90]. Dysregulation of interactions between immune cells, keratinocytes, and environmental factors, including microbiota, has been implicated in pathogenesis of psoriasis [90]. Unlike the inflammatory diseases discussed above, the role of microbial alterations in psoriasis is much less understood. Up to date, several reports described disturbances in microbiota composition specific to psoriatic skin. However, these reports vary in defining microbial signature associated with this disease. Lack of the common, well-defined microbial pattern in psoriasis individuals, could be at least partly attributed to differences in experimental design reported in these studies, including sampling methods, sequencing methods, as well as sampling sites that involve both sebaceous and dry skin regions [91].

Taxonomic variety of psoriasis-associated microbiota is still under debate. For example, an increased heterogeneity in psoriasis-associated microbiota was observed when compared to healthy skin [10]. Moreover, psoriatic skin was reported to be readily colonized by poorly characterized, unknown microbes [11]. In contrast, an opposite correlation was also reported, where decreased bacterial diversity was observed in psoriatic skin compared to normal skin [92, 93]. Other studies did not show significant difference among skin biopsies obtained from healthy donors and individuals with psoriasis [94]. When taking different skin microenvironments into account, species diversity was reduced in the sebum-rich areas of psoriatic lesions when compared to healthy skin, with dry sites bore similar microbial variety [11].

Dysbiosis of the psoriasis-associated skin microbiome was more apparent at the genus level. The main changes involved *Staphylococcus, Streptococcus* and *Cutibacterium* burden [10, 92–94]. *Streptococcus* species were found to be overrepresented in lesional skin. Additionally, the ratio of *Streptococcus/*cutibacteria was also elevated in psoriatic patients [93, 94]. High abundance of *C. acnes* in healthy skin was shown to negatively correlate with abundance of several other bacteria, including various staphylococci. Some studies highlight the role of *Staphylococcus* spp. in psoriasis [10, 89]. It was reported that *S. aureus* and two additional *Staphylococcus* species, *S. sciuri* and *S. pettenkoferi* were more abundant in psoriatic patients, whereas *S. epidermidis* preferentially colonized healthy skin [10]. Overrepresentation of *S. aureus* in skin collected from psoriasis patients suggests a potential link of these bacteria with pathogenesis of psoriasis. This is supported by the reports that in vitro exposition of T cells to *S. aureus*-derived antigens promotes associated with psoriasis Th17-based immune response [95]. Given important role of Th17 lymphocytes in the immunopathogenesis of psoriasis [89] and a recent finding that *S. aureus* can trigger Th17 polarization of CD4^+^ T cells during skin colonization in newborn mice, a potential involvement of *S. aureus* in pathogenesis of psoriasis is plausible [10]. To conclude, the microbial shift that occurs in psoriasis is associated with decrease in beneficial species at the expense of less friendly ones, like *S. aureus*. Nevertheless, specific impact of these species on PS development is yet to be fully elucidated.

## Conclusion

Novel approaches to studying microbial communities, especially focused on metagenomics, helped to shed a new light onto microbiome-host interactions. As discussed in previous sections, skin disorders are associated with modifications in the cutaneous microbiota patterns. It has become apparent that classifying specific bacterial species as a pathogen or a commensal depends on the context. Interactions with host cells or strain-specific differences can control microbe switch from passive to downright aggressive behavior. Using transcriptomic analysis, as well as re-analysis, can help to elucidate which genes are important to chronic inflammatory diseases. In conclusion, while a great progress has been made in skin microbiome research, specific microbial signatures that can mechanistically explain pathogenic skin conditions or serve as diagnostic markers in inflammatory skin disorders remain to be fully characterized. However, classical in vitro and in vivo studies supplemented by vast data from transcriptomic and NGS can rapidly facilitate understanding the role of skin microbiome in the development of chronic skin inflammatory diseases.
